# The impact of community containment implementation timing on the spread of COVID-19: A simulation study

**DOI:** 10.12688/f1000research.24156.1

**Published:** 2020-05-27

**Authors:** Attayeb Mohsen, Ahmed Alarabi

**Affiliations:** 1 Laboratory of Bioinformatics, Artificial Intelligence Center for Health and Biomedical Research (ArCHER). National Institutes of Biomedical Innovation, Health and Nutrition., Ibaraki city, Osaka, 567-0085, Japan; 2 Department of Pharmaceutical Sciences, Irma Lerma Rangel College of Pharmacy, Texas A&M University, Kingsville, Texas, 78363, USA

**Keywords:** Simulation, Outbreak, COVID-19, Community containment, Social distancing

## Abstract

**Background: **Community containment is one of the common methods used to mitigate infectious disease outbreaks. The effectiveness of such a method depends on how strictly it is applied and the timing of its implementation. An early start and being strict is very effective; however, at the same time, it impacts freedom and economic opportunity. Here we created a simulation model to understand the effect of the starting day of community containment on the ﬁnal outcome, that is, the number of those infected, hospitalized and those that died, as we followed the dynamics of COVID-19 pandemic.

**Methods: **We used a stochastic recursive simulation method to apply disease outbreak dynamics measures of COVID-19 as an example to simulate disease spread. Parameters are allowed to be randomly assigned between higher and lower values obtained from published COVID-19 literature.

**Results: **We simulated the dynamics of COVID-19 spread, calculated the number of active infections, hospitalizations and deaths as the outcome of our simulation and compared these results with real world data. We also represented the details of the spread in a network graph structure, and shared the code for the simulation model to be used for examining other variables.

**Conclusions: **Early implementation of community containment has a big impact on the ﬁnal outcome of an outbreak.

## Introduction

As a public health tool, community containment measures are employed to curb/slow the spread of infectious disease outbreaks inside communities, regions, and cities. It is considered a last resort when all other tools such as case-isolation and quarantine fail to yield the required results ^1^. Community containment can be achieved by applying a number of measures, which can vary from social distancing (by closing schools, cancelling big population events, or travel ban) to a complete shutdown/lock-down of entire region or city ^1^. The main goal of community containment measures is to lower the pace and the extent of an outbreak, or “flattening the curve” ^2^, which helps in preventing bottlenecks in the healthcare system. During outbreak acceleration phase, the number of people needing hospitalization and critical care grows exponentially and that can overwhelm healthcare system and lead to sub-optimal treatment, which eventually is translated to higher morbidity and mortality.

The social distancing policies implemented during pandemic are not without a cost; these measures lead to an economic slowdown. To manage that, authorities might push for relaxation of the containment methods to improve economic opportunity, a step that might be too early and could lead to increase in the spread of infection. Therefore, authorities must maintain a delicate balance between the country economic performance and the health of its citizens ^3^.

To this end, simulation is a commonly used method to inform our understanding of the impact of several factors/variables on the dynamics of infectious disease. The different types of simulation methods and algorithms are reviewed by Siettos *et al*. ^4^. Recently, many studies have been published to simulate the dynamic of COVID-19. One particular paper ^5^ examined the effect of case isolation. However, The results of simulating the timing for community containment, to our knowledge, have not been published yet. In this paper, we aim to show this using a recursive simulation model of COVID-19 ^6, 7^ infection spread. Anderson *et al*. ^2^ published a detailed commentary discussing the dynamics of COVID-19 spread and how its spread can be mitigated, which we have used as the basis of our simulation. Although this simulation can be tuned to examine the effect of multiple factors, we examined/showed only the impact of the timing of community containment implementation. Moreover, we presented the spread in a graph network structure to facilitate further investigation.

## Methods

This simulation started by assuming the presence of one patient in equally infection-susceptible community and remained infectious for 6 days. Every day he came to contact with random number of people between 10 to 15, with 3% probability for each one of them to be infected (secondary case). Each secondary case get into incubation period with random assigned length between 3 to 14 days and started to be contagious 1 or 2 days before the end of that period, all other possibilities are randomly modeled to resemble COVID-19 dynamics, the possible outcomes are diagnosed cases, hospitalizations (20%), deaths (6%) or cures ^2, 7^.

To imitate a real life situation, the patient is counted (active case) only after being diagnosed, there is a possibility for a patient to be diagnosed even before developing any symptoms (end of incubation period).

To simulate community containment measures, we simplify the effect to reduction of number of contacts. Originally the number of contacts are set to be 10–15 per day, We set two levels of containment measures: loose, where the number of contacts is reduced from 10–15 to 5–10 per day (40% reduction); strict, where the number of contacts is reduced to 2–5 per day (~ 72% reduction). We calculated the effect of starting community containment (35, 45, 55, 65, 75, and 85) days after first patient diagnosis. This simulation continued for 100 days. And we recorded the details of infected cases and their origin of infection, so they can be used to build a network graph if other observations are intended like simulating the effect of big transmitters, however, we did not consider any of them in this short article.

We tested the effect of community containment relaxation by going back to normal number of contacts after one month in the case of starting community containment after 45 days of first patient diagnosis. Moreover, we compared our simulation result with real data from Our World in Data ^8^. We compared the number of cases of India and San Marino from the report of first case to the date we did the analysis on May 10th, 2020.

We used Python programming language (version 3.7.6), with the following packages: Networkx 2.4 for network analysis, json 2.0.9 and gzip packages for data storage, pandas 1.0.1 and numpy 1.18.4 for data processing and Matplotlib 3.1.3 for visualisation.

## Results

### Simulation model

The created simulation code is available at www.github.com/attayeb/corona_simulation/ ^9^. It is a simple python script that takes input of two items, the details of first patient and the simulation parameters which need to be inserted in form of a python dictionary. This code can be easily modified to fulfil the requirement of wide spectrum of parameters, like the number of contacts, quarantine measures, hospitalization, and death rates.

### Simulation of community containment

Because of the stochastic nature of our simulation, and to get the most reliable result of our work, we did the simulation 100 times, and we calculated the median, we choose median to reduce the effect of outliers.

**Figure 1.**
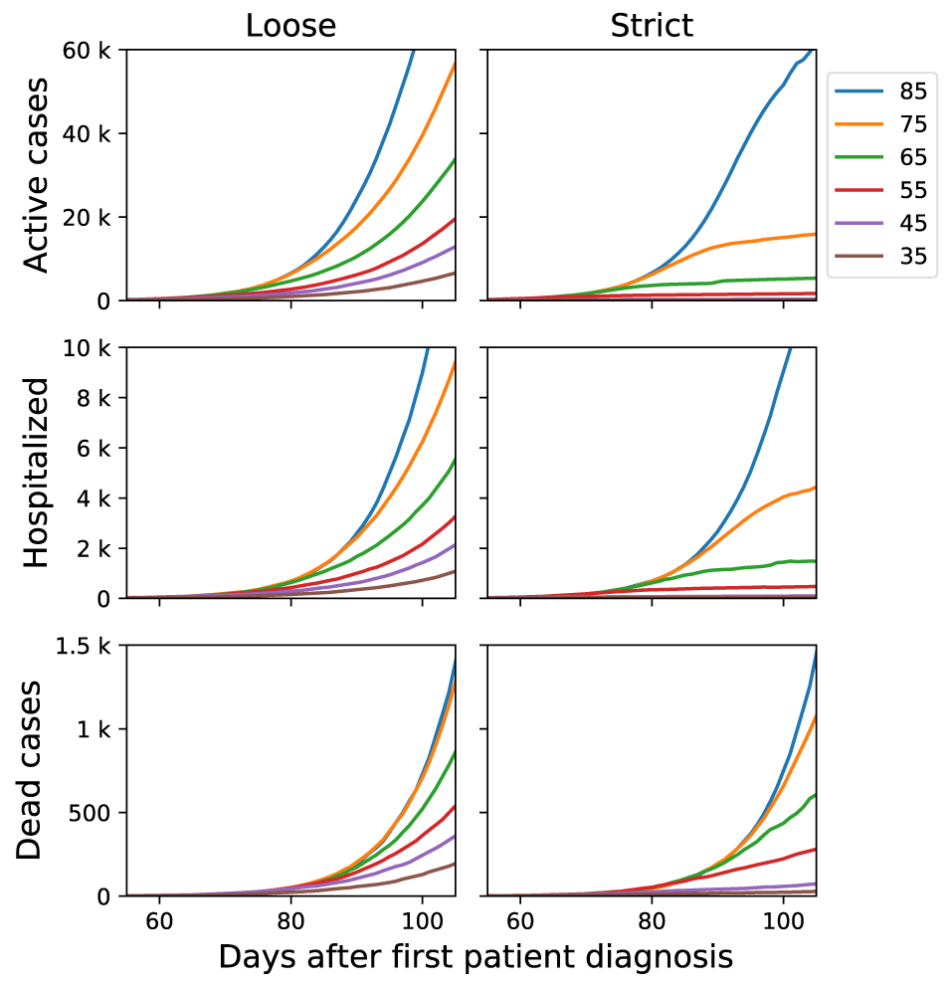
Comparing the numbers of active cases, hospitalized, and deaths of loose versus strict scenario. The x-axis shows the day after first patient diagnosis, colored lines correspond to the day when community containment starts.

**Figure 2.**
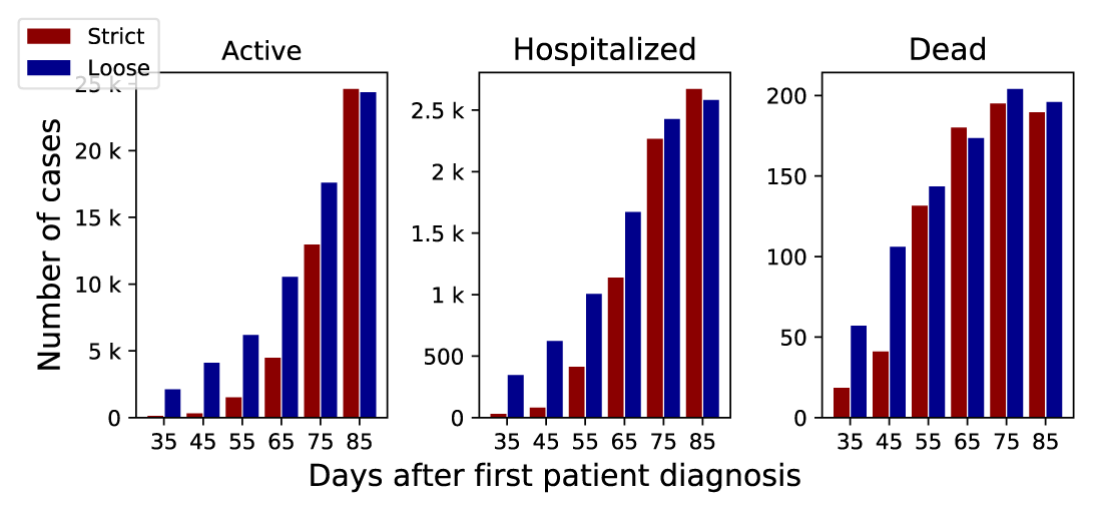
A summary of simulation results at day 90. The x-axis represents the start day of the community containment measures, y-axis represents the number of cases.

The number of active cases ( Figure 1), shows how implementing community containment measures earlier after first patient diagnosis has greater effect. For example, starting community containment measures 10 days earlier (65 compared to 75) leads to 60% reduction in number of active cases at day 90. Same behaviour can also be seen with the number of hospitalized cases ( Figure 2). The effect on number of dead cases are more prominent at day 45 compared to 65 or later because longer time is required for death to happen in comparison to diagnosis and hospitalization.

**Figure 3.**
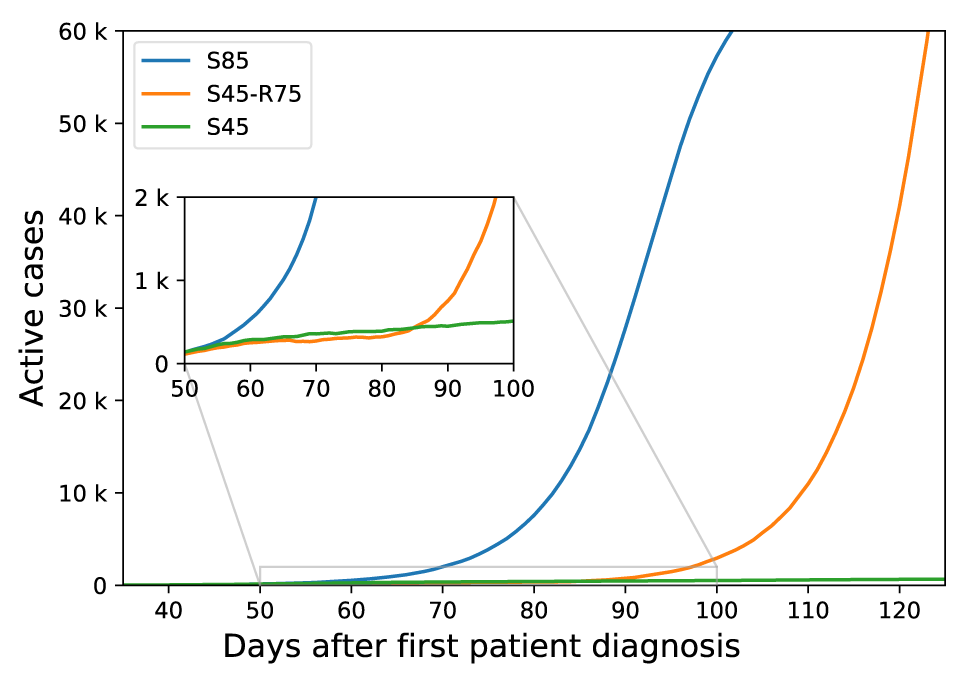
The effect of relaxing community containment measures on the number of active cases. S85: starting community containment measures at day 85, S45-R75: starting community containment at day 45 with relaxation at day 75, S45: starting community containment at day 45 without relaxation.

To show the effect of community containment measures relaxation, we simulated going back to usual number of contacts after one month from starting as an example. Figure 3 showed that, after one month of community containment measures, we go back to the same curve slope compared to the original curve without applying any measures. Which means that relaxing community containment after one month of applying them is delaying the infection spread without curve flattening. Our model can be used for further consideration of the effect of duration of community containment.

**Figure 4.**
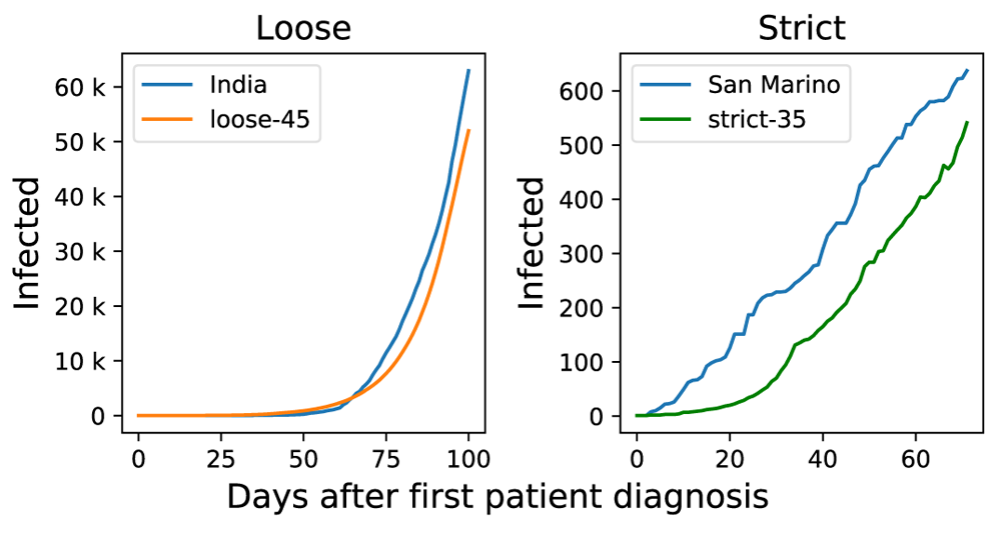
Simulation results compared to real world statistics. left side panel shows loose measures in India corresponds to loose-45 (applying loose measures 45 days after first patient diagnosis) model, while right side panel shows strict-35 model (applying strict measures 35 days after the diagnosis of first patient) model in comparison with San Marino statistics.

We also compared our model results to real world data. Figure 4 shows that our loose-45 model is comparable to India spread. In India, the first case was recorded on January 30th, and major lockdown measures were introduced by the government on March 22nd, 50 days after first patient diagnosis. Meanwhile our strict-35 model is similar to the situation in San Marino, which had the first case reported on February 27th and on March 14th a major lockdown started.

### Network analysis

**Figure 5.**
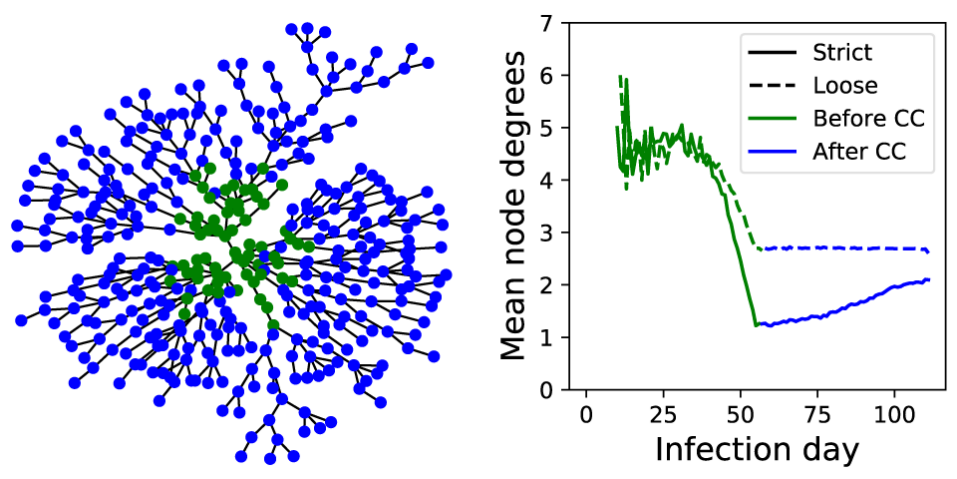
Simulation network visualization and analysis. Left panel shows miniature example of built network graph for strict-55 model. Green colored nodes are the cases infected prior to introduction of community containment. Right panel shows the mean of node degrees, which corresponds to the number of cases got infected per patient. CC, community containment.

To show the effect of community containment on the number of COVID-19 infections transmitted by every single patient, we built a spread network graph ( Figure 5). Nodes represent infected cases and edges represent infection transmission. Therefore, the calculation of nodes degree over time reflects the transmission rate for each patient. This figure shows that strict measures leads to greater reduction in number of new infected patients per case compared to loose measures and before community containment. This network can be further used to study the effect of other factors such as the incubation period and self-quarantine.

## Discussion

COVID-19 is a highly contagious disease with a devastating morbidity and mortality burden ^10^, which led to a debate/uncertainty about the likelihood of its spread in the community ^11^. It is estimated that 20% of COVID-19-infected people suffer severe clinical complications that need urgent and complex treatment, and therefore have the potential to overwhelm health care facilities such as hospitals ^11^. Overburdened hospitals effect might also be compounded by the shortage of trained health care professionals who deliver treatment, and hospital staff who organize the workflow of the day-to-day operation ^12^. Collectively, this can lead to poor quality of care and in some cases a complete exhaustion of the health care system, similar to what happened in the Italy ^13^. With this in mind, community containment methods such as social distancing had been employed to slow down the spread of COVID-19 in affected communities and consequently relieve overburdened healthcare systems and reduce the number of deaths.

For this purpose, in this simulation study, we have shown that early implementation of social distancing measures (reduction in the number of contacts of people as a general rule) in a whole society has a great impact on the numbers of infections, hospitalizations and deaths as a result of COVID-19 infection. In addition, we demonstrated that early relaxation of social distancing leads to a rebound increase in the number of active cases in the community without curve flattening. We achieved these results by stochastic simulation modelling of the spread with simulation assumptions determined by following the COVID-19 published literature ^2^. We have done the simulation 100 times for each parameter set and calculated the median to reduce the impact of randomization and outliers on the final result.

One major limitation in our model is the fact that it does not consider the Susceptible, Infected and Removed (SIR) model; we purposefully did that because we are targeting the impact of early application of the measures rather than the extended effect. Moreover, there is no clear conclusion regarding the effectiveness of post-infection COVID-19 immunity ^14^ for SIR model to rely upon. We also considered that applying the measures at a later stage (75 or 85 days) is not practically possible, assuming that the population will follow self-quarantine guidelines if wide disease spread happens; however, we are still showing these results for comparison as extreme case when all protective measures are ignored. We also did not consider the effect of self-quarantine or case isolation strategy if the patient was not hospitalized, because our aim is to show the impact of global society rather than individual preventive measures which other simulation studies have considered ^5^. However, small modifications of our script simulation variables input could be done to simulate such effects.

During this manuscript preparation, similar results were reported by Matrajt *et al*. ^15^. They followed age-stratified compartments similar to the population of Seattle metropolitan area. Our model in comparison did not consider age distribution; however, one important addition of our model is the presentation of the simulation results in graph structure. Our simulation also was limited to the first 100 days of the pandemic without assuming the total community population. Finally, our simulation was compared to an actual COVID-19 containment measures implemented in San Marino and India, which gives more robustness to our model.

## Conclusions

This simulation shows that the early implementation of social distancing and reducing the number of contacts per day reduces the number of hospitalized cases, deaths and number of infected people. Furthermore, early relaxation of social distancing is associated with rebound increase in the number of active cases in the community. This study can provide some evidence that can help increase awareness among health care professionals, policy makers, and general public about the importance of early implementation of community containment measures such as social distancing during pandemics.

## Data availability

### Underlying data

All data underlying the results are available as part of the article and no additional source data are required.

### Extended data

**Source code and input data for the simulation available at:** https://github.com/attayeb/corona_simulation/

**Archived source code at time of publication:** https://doi.org/10.5281/zenodo.3835945 ^9^.

**License:** BSD 3-Clause License.
