# Supplementary material for: The impact of community containment implementation timing on the spread of COVID-19: A simulation study
Source: F1000Res. 2020 May 27;9:452. [Version 1] doi: 10.12688/f1000research.24156.1 (PMC7463296; doi:10.12688/f1000research.24156.1)
Supplement: Peer review history

# Peer review: Reviewer response for version 1

**DOI:** 10.5256/f1000research.26644.r70458

**Published:** 2020-09-21

**Authors:** Mahmoud Heshmat

**Affiliations:** 1 Department of Mechanical Engineering, Faculty of Engineering, Assiut University, Assiut, Egypt

In the manuscript, the authors developed a simulation model to study the impact of community containment implementation timing on the spread of COVID-19. The manuscript can be accepted provided that the following issues are solved.

1. In the abstract, the results section should be highlighted in a better way. For example, the first sentence is part of methodology not result.
2. You mentioned in the conclusions in the abstract that Early implementation of community containment has a big impact on the final outcome of an outbreak. How much does that impact? How do you measure it?
3. The authors used the work in references [3, 6, 7] from the literature, is it a considerable contribution? The authors should clearly illustrate the contribution.
4. The literature review is limited, the authors must expand it by mention similar cases of using simulation as a tool in healthcare in general and COVID 19 in particular.
  Examples: ^1^^,^^2^
5. The manuscript should be revised with respect to grammatical and structure issues. I am giving examples below:
  Abstract: The first sentence is very short, I suggest being merged with second one or moved to the introduction.
  Introduction: Paragraph 3 Line 7: “However, The results of simulating the timing for community containment, to our knowledge, have not been published yet.”
  Line 4: the structure should be enhanced.
  Paragraph 2: Heshmat et al. takes….
  Page 4, second paragraph: “….which we studied only the treatment unit..” not clear structure.
  Page 4, last line in the second paragraph: c in Chemotherapy should be small.
  Conclusion: This simulation shows that the early implementation of social distancing and reducing the number of contacts per day reduces the number of hospitalized cases, deaths and number of infected people.

Is the work clearly and accurately presented and does it cite the current literature?

Partly

If applicable, is the statistical analysis and its interpretation appropriate?

No

Are all the source data underlying the results available to ensure full reproducibility?

Partly

Is the study design appropriate and is the work technically sound?

Yes

Are the conclusions drawn adequately supported by the results?

Yes

Are sufficient details of methods and analysis provided to allow replication by others?

Yes

Reviewer Expertise:

Simulation.

I confirm that I have read this submission and believe that I have an appropriate level of expertise to confirm that it is of an acceptable scientific standard, however I have significant reservations, as outlined above.

## References

[1] : A system dynamics-based decision support model for chemotherapy planning. *Journal of Simulation*.2018;12(4) : 10.1057/s41273-017-0059-8 283-294 10.1057/s41273-017-0059-8

[2] : Modeling Simulation of COVID-19 in Indonesia based on Early Endemic Data. *Communication in Biomathematical Sciences*.2020; 10.5614/cbms.2020.3.1.1 10.5614/cbms.2020.3.1.1

# Peer review: Reviewer response for version 1

**DOI:** 10.5256/f1000research.26644.r67222

**Published:** 2020-09-01

**Authors:** Indumathi Venkatachalam

**Affiliations:** 1 Department of Infection Prevention and Epidemiology, Singapore General Hospital, Singapore, Singapore; 2 Department of Infectious Diseases, Singapore General Hospital, Singapore, Singapore

In this global Covid-19 pandemic, all societal sectors have been impacted. As the pandemic spread across the globe, countries have responded differently, some instituting community containment measures early while others awaiting in anticipation for development of protective herd immunity. Each strategy is fraught with deep uncertainties and unprecedented far reaching consequences.

 Mohsen and Alarabi present a simulation study that predicts the impact of community containment measures on the numbers of Covid-19 cases, hospitalizations and deaths. They assess, (1) strict versus loose containment measures and (2) time from first case to implementation of these measures. They then compare their models to real data from India and San Marino, from report of first case to date of analysis on May 10 ^th^, 2020.

 Though the results have to be interpreted in light of the assumptions such as infectious period of 6 days, 3% probability of being infected once exposed, 20% hospitalization and 6% death rates from Covid-19, the overall finding that early implementation of community containment measures may be more important than the strictness of these measures is interesting and worth exploring further.

 Results in Figure 2 can be elaborated further; the results highlight the time to initiation of community containment measures has a greater impact than the type of measures. Looser measures early on have better outcomes, and when interventions are implemented later on, stricter measures don't seem to have much benefit over looser measures.

 The validity of these findings have potential to impact policy and outcomes for subsequent waves of this pandemic.

Is the work clearly and accurately presented and does it cite the current literature?

Yes

If applicable, is the statistical analysis and its interpretation appropriate?

Partly

Are all the source data underlying the results available to ensure full reproducibility?

Yes

Is the study design appropriate and is the work technically sound?

Yes

Are the conclusions drawn adequately supported by the results?

Yes

Are sufficient details of methods and analysis provided to allow replication by others?

Yes

Reviewer Expertise:

Infectious disease epidemiology, including emerging infectious diseases and multi-drug resistant bacterial infections.

I confirm that I have read this submission and believe that I have an appropriate level of expertise to confirm that it is of an acceptable scientific standard.

# Peer review: Reviewer response for version 1

**DOI:** 10.5256/f1000research.26644.r65343

**Published:** 2020-07-07

**Authors:** Salem I.M Alkoshi

**Affiliations:** 1 Department of Public Health, Faculty of Public Health & Nursing, Al Asmarya Islamic University, Zliten, Libya

it is a good work with sufficient data analyzed. It was good written, but I have minor comments which should be done for final approval:

1. The result in the abstract should include some numbers and clear results rather than narrative. this will strengthen the abstract.
2. Please provide more explanation about 85 days in fig 1 page 4, it seems to be same results in loose and strict, Please clear it in result part or discussion part.

Great work and hope all the best to authors.

Is the work clearly and accurately presented and does it cite the current literature?

Yes

If applicable, is the statistical analysis and its interpretation appropriate?

Yes

Are all the source data underlying the results available to ensure full reproducibility?

Yes

Is the study design appropriate and is the work technically sound?

Yes

Are the conclusions drawn adequately supported by the results?

Yes

Are sufficient details of methods and analysis provided to allow replication by others?

Yes

Reviewer Expertise:

Public Health

I confirm that I have read this submission and believe that I have an appropriate level of expertise to confirm that it is of an acceptable scientific standard.
